# Relaxation Oscillation with Picosecond Spikes in a Conjugated Polymer Laser

**DOI:** 10.3390/polym8100364

**Published:** 2016-10-14

**Authors:** Wafa Musa Mujamammi, Saradh Prasad, Mohamad S. AlSalhi, Vadivel Masilamani

**Affiliations:** 1 Department of Physics and Astronomy, College of Science, King Saud University, Riyadh 11451, Saudi Arabia; walmujammi@ksu.edu.sa (W.M.M.); srajendra@ksu.edu.sa (S.P.); malsalhi@ksu.edu.sa (M.S.A.); 2 Research Chair on Laser Diagnosis of Cancers, College of Science, King Saud University, Riyadh 11451, Saudi Arabia

**Keywords:** conjugated polymer (PFO-*co*-MEH-PPV), green laser, relaxation oscillations, amplified spontaneous emission spectra (ASE), picoseconds spikes

## Abstract

Optically pumped conjugated polymer lasers are good competitors for dye lasers, often complementing and occasionally replacing them. This new type of laser material has broad bandwidths and high optical gains comparable to conventional laser dyes. Since the Stokes’ shift is unusually large, the conjugated polymer has a potential for high power laser action, facilitated by high concentration. This paper reports the results of a new conjugated polymer, the poly[(9,9-dioctyl-2,7-divinylenefluorenylene)-*alt*-*co*-{2-methoxy-5-(2-ethylhexyloxy)-1,4-phenylene}](PFO-*co*-MEH-PPV) material, working in the green region. Also discussed are the spectral and temporal features of the amplified spontaneous emissions (ASE) from the conjugated polymer PFO-*co*-MEH-PPV in a few solvents. When pumped by the third harmonic of the Nd:YAG laser of 10 ns pulse width, the time-resolved spectra of the ASE show relaxation oscillations and spikes of 600 ps pulses. To the best of our knowledge, this is the first report on relaxation oscillations in conjugated-polymer lasers.

## 1. Introduction

Conjugated polymers consist of alternating single and multiple (mostly double) carbon-carbon bonds. The simplest is polyacetylene, consisting of a string of carbon atoms joined by alternating single and multiple bonds [1,2]. In 1977, it was discovered that doped conjugated polymers could act as semiconductors [3]. Alan Heeger, Alan MacDiarmid, and Hideki Shirakawa shared the Nobel Prize in Chemistry in 2000, for the discovery and development of conductive polymers. Their work focused on polyacetylene, which was first used in a photovoltaic cell in 1982 [4]. Such conjugated polymers have a high degree of technological significance; they are the examples of organic semiconductors interacting strongly with light and hence useable in a wide range of optoelectronic devices [5,6,7,8].

The interactions of light with conjugated polymers has important scientific significance. For the conjugation lengths typical of these systems, the HOMO-LUMO energy gap approaches that accessible by the visible (450–800 nm) and near-ultraviolet (350–450 nm) regions of the electromagnetic spectrum. In a series of earlier papers, this group has reported the spectral and the laser properties of some of the important conjugated polymer laser [9,10,11,12].

To understand the photophysical properties of conjugated-polymer materials in general, their time-resolved photoluminescence is measured by exciting the luminescence from a sample with a pulsed light source, and then measuring the subsequent decay of the photoluminescence (PL) as a function of time. A wide variety of experimental configurations can accomplish this. Most experiments excite the sample with a pulsed laser source, and detect the PL with a photodiode, a streak camera, or a photomultiplier tube set up with up-conversion or single-photon counting. The system response time, wavelength range, sensitivity, operational difficulty, and cost vary widely for each configuration [13].

Very short time scales characterize many of the fundamental photophysical processes of conjugated polymers. For example, the fluorescence lifetime of a conjugated polymer is typically of the order of 1 ns. Exciton energy transfer occurs with a period between 10 and 100 ps, with the thermalization of the excess vibrational energy on a polymer chain occurring over 1000 ps. Consequently, time-resolved laser spectroscopy leads the experimental investigations into the photophysical properties of conjugated polymers, with the ability to monitor events of timescales as short as 100 ps [14,15,16].

The damped relaxation oscillations, frequently observed in the Nd YAG or ruby lasers, have been fully explained by simple rate equations. These oscillations arise from the interplay between the photon flex and the atomic or molecular state population inversion. With pulsed dye lasers, such a transient oscillatory occurrence is possible with short cavities [17]. With a 1 cm long dye laser with a cavity, well-developed relaxation oscillations were reported in the case of 10 ns pumping from an N2-1aser and it was shown by the authors that the near-threshold pumping can be used to produce a single peak subnanosecond pulse. Pulses as short as 10 ps were observed by using 600 ps pumping pulses in a 40 µm-long cavity [18]. Such a method suffers from the inherent instability of the near-threshold operation.

Relaxation oscillation was observed in a laser system made of neodymium organo-metal complex (Nd(TTA)3phen) embedded on a polymer matrix (6-FDA/UVR and EHPE). Oscillation occured as pulse few microseconds [19].

Dykstra et al. provided a model and experimentally verified that PPV-family conjugated polymers with different conformation subunits can electronically couple to neighboring subunits, and the relaxation of these exciton states occurs on ultrafast time scales [20]. The time-resolved emission spectra with a 200 fs resolution, enabled to understand various photo-physics mechanism including the relaxation dynamics of pristine regioregular P3HT in dilute chlorobenzene solution [21].

In this paper, we present evidence for relaxation oscillation arising from the dynamic interaction between the photon flux and population inversion existing in the conjugated polymer.

## 2. Experimental

**Figure 1:**
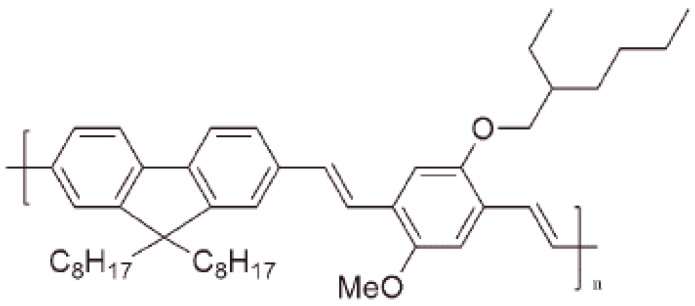
The molecular structure of the conjugated-polymer PFO-*co*-MEH-PPV (green polymer, GP).

For this study, a green polymer poly[(9,9-dioctyl-2,7-divinylenefluorenylene)-*alt*-*co*-{2-methoxy-5-(2-ethylhexyloxy)-1,4-phenylene}] (PFO-*co*-MEH-PPV or GP) was purchased from the American Dye Source (Baie-d’Urfé, QC, Canada) and used as such. It is a pure substance with an average molecular mass equal to 256,000. The molecular structure is shown in Figure 1. The photoluminescence and laser properties of this polymer conjugation has not been studied before.

The absorption and the fluorescence spectra were measured for the GP in different concentrations. The spectra for the solutions were measured using a small quartz cuvette with dimensions of 1 × 1 × 4 cm and an optical path length of 1 cm. UV–Vis absorption spectra were obtained using a Perkin Elmer spectrophotometer and the fluorescence spectra measured on a Perkin Elmer LS55 spectrofluorometer (London, UK). 

The third harmonic (355 nm) of an Nd:YAG laser (Brilliant B of Quantel, Les Ulis Cedex, France), with a pulse width of 10 ns, was used as the excitation source. The UV laser was focused using a quartz cylindrical lens (plano-convex) of focal length 5 cm. This was used to transverse excite the GP solution taken in a four-sided polished quartz cell and kept canted to avoid feedback; see reference [8] for more details. In case of longitudinal pumping, the cylindrical lens was replaced by a quatz spherical lens of focal length 5 cm. At optimum values of the pump power and the concentration of the GP, amplified spontaneous emissions (ASE) could be achieved as a beam coming out in a cone of light. This was fed to an ultrafast Si-photodetector (UPD) [ALPHALAS GmbH (Goettingen, Germany): P/N:UPD-200-UP, Rise time: 120 ps] and sampling oscilloscope [Textronix (Beaverton, OR, USA): DPO4104B-L, Digital Phosphor: 1 GHz, 5 GS/s, 4 channels] with a combined time resolution of 200 ps. This combination was used to monitor the temporal properties of the ASE pulses collected in parallel by a fiber optic cable and fed to a spectrograph [Acton SP-2360 Imaging Spectrograph (Trenton, NJ, USA)] attached to an ICCD camera [Princeton Instruments PI-MAX4: 1024 × 256 pixel, 25 mm-RB Intensified Camera System, (Trenton, NJ, USA)], to obtain a 3D display of the spectral and temporal features of the ASE.

## 3. Result and Discussion

### 3.1. Spectral Properties

**Figure 2:**
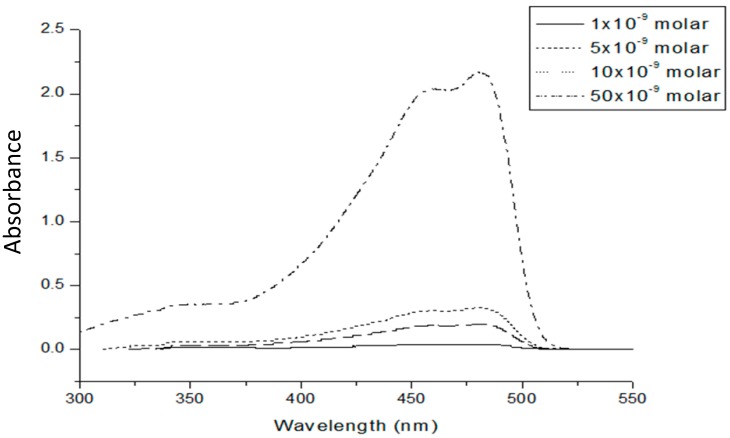
Absorption spectra of GP in toluene for concentration ranging from 1 nanomolar to 50 nanomolar.

The spectral and temporal features of the laser from the conjugated polymer GP could be understood only from a study of the absorption and the emission spectra of the polymer material. The absorption spectra of the conjugated polymer (PFO-*co*-MEH-PPV) was obtained by dissolving 1 mg of the polymer in 10 mL of solvent toluene. By serial dilution, solutions of different concentrations were obtained for UV–Vis absorption. From Figure 2, we had noted that the absorption spectral features were almost the same for the concentrations used, although the optical density increased with the rise in concentration. This indicates the absence of any dimer (aggregation) in the ground state for all the concentrations used. We noted that there were two peaks of absorption at two wavelengths, one at 450 nm and the other at 480 nm, which were repeated for all concentrations, but the main peak was always at 480 nm.

**Figure 3:**
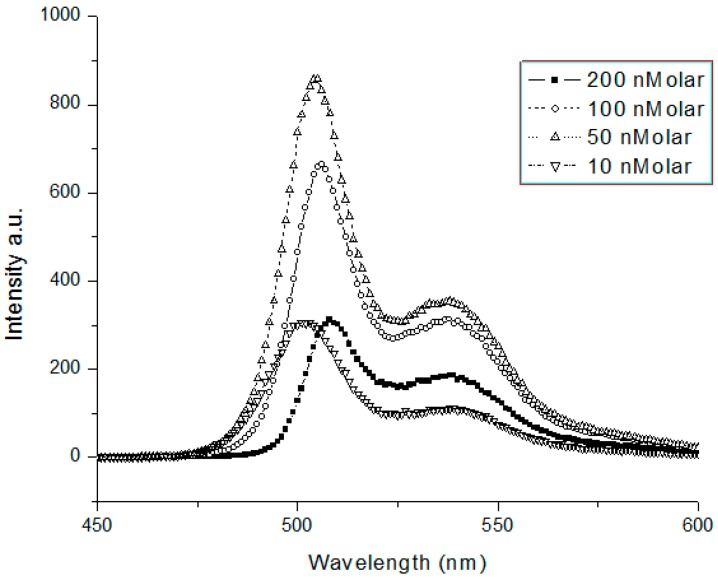
Fluorescence spectra of GP in toluene for concentration ranging from 10 nanomolar to 200 nanomolar.

The fluorescence spectra results showed that there was only one peak around 505 nm with a shoulder at 540 nm, and the shape of the fluorescence spectra did not change for different wavelengths of excitation. In addition, the shape of fluorescence spectra did not change for concentrations ranging from 10 to 200 nM as shown in Figure 3.

We noted that the intensity started to increase from 10 nM to the maximum value in 50 nM and decreased thereafter. The critical concentration for this solution to fall in fluorescence was 50 nM. This could be attributed to quenching by collision of the molecules in excited states because of the very high molecular mass (256,000) of the GP. The quantum yield of the conjugated polymer in toluene was more than 0.49, as determined by the conventional method with coumarin 500 as the standard [22]. This conjugated polymer GP was soluble only in very few solvents such as toluene and benzene, and their spectral features (for a concentration of 500 nM) are listed in Table 1.

**Figure 4:**
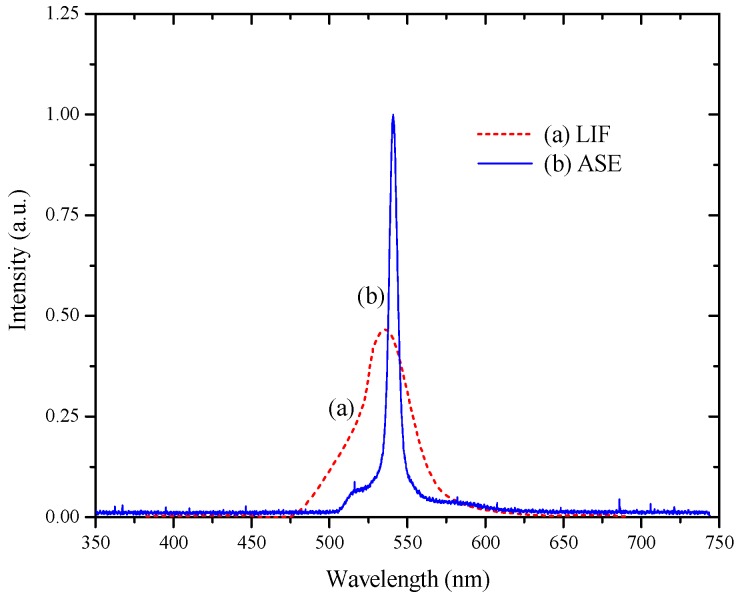
(**a**) Laser induced fluorescence (LIF) for high concentration (1 µM) at low pump power (1 mJ) and (**b**) amplified spontaneous emission (ASE) for the same concentration at 2.3 mJ pump power.

The laser induced fluorescence (LIF) spectrum was obtained for this conjugated-polymer dissolved in toluene at concentrations of 100 nM and 1 µM. These solutions were excited by the third harmonic of an Nd:YAG laser (355 nm). The LIF peaks were ~535 nm with a shoulder ~510 nm, as shown in Figure 4a. For the higher concentration (1 µM) and low pump energy, we recorded a strong LIF spectrum with only one peak at 540 nm, as shown in Figure 4. This was due to a strong overlap of the absorption and the emission bands. For higher pump energy (4 mJ), the ASE observed had a peak near 540 nm with a narrow spectral bandwidth of 6 nm (FWHM), as shown in Figure 4b. This peak coincided with the maximum of the fluorescence emission spectrum at this concentration, as shown in Figure 4a.

### 3.2. Temporal Features of Conjugated Polymer Laser

Several studies of spectral features for conjugated polymers such as the PFO, and the MEH-PPV, have produced laser from the dimer, excimer, and double excimer state [9,10,11,12]. However, no one has conducted a time-domain study for any of them. We have presented here the excited state behavior in the subnanosecond time scale, typical of a host of the conjugated polymers.

**Figure 5:**
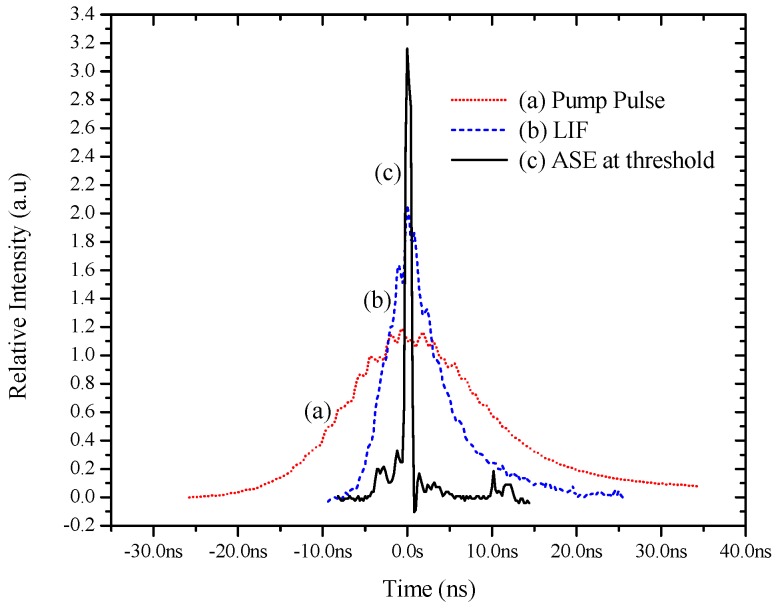
Shows the temporal profile of (**a**) pump pulse; (**b**) LIF and (**c**) ASE, with a full width half maximum (FWHM) of 10, 5 and 1 ns respectively.

Figure 5a shows the temporal profile of the bell shaped pump pulse (third harmonic of the Nd:YAG laser) with a FWHM of 10 ns. Figure 5b shows the time evolution of the LIF from the conjugated polymer with a smooth sharp pulse of 4 ns (FWHM). Figure 5c represents the ASE pulse obtained when the pump energy goes above the threshold (2.3 mJ). One can see the characteristic time narrowing of the ASE pulse to the 1 ns width. That is, when the LIF becomes ASE, the pulse width narrows down to 1 ns from 5 ns. This can be compared with the earlier Figure 4, where the LIF has a spectral width of 30 nm. However, the ASE had a spectral width of 6 nm, a spectral narrowing by a factor of 5. These are two characteristic manifestations of the stimulated emission dominating over spontaneous emission with the onset of the ASE.

**Figure 6:**
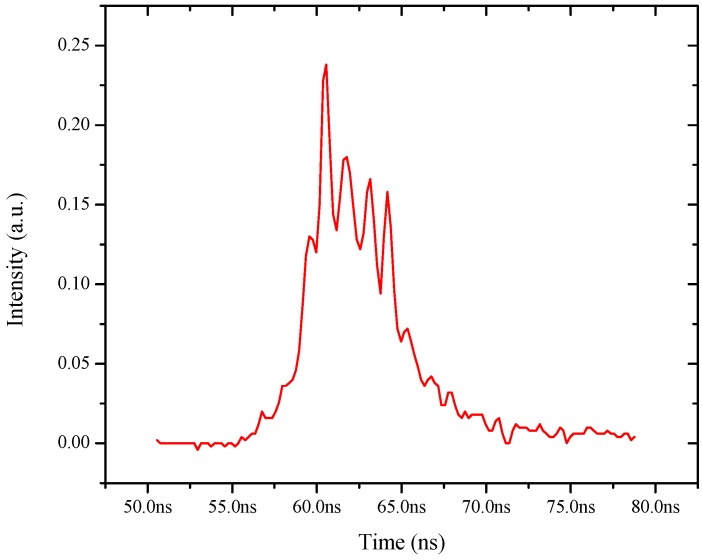
Shows the temporal profile of ASE for pump pulse energy (4 mJ) well above threshold, one could see the emission become jittery with spikes of 600 ps attributed the relaxation oscillation.

**Figure 7:**
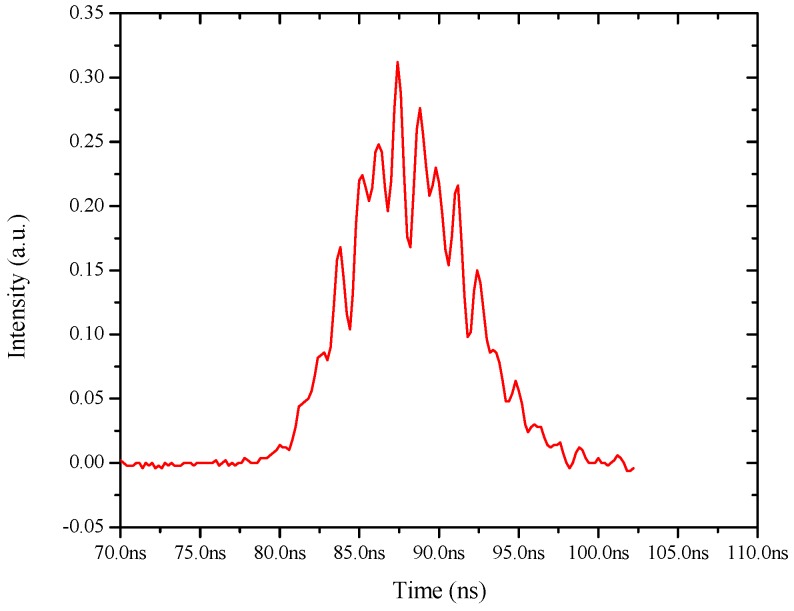
Shows the temporal oscillation trace of another relaxation oscillation pulse, indication of non-reproducible spikes.

Figure 6 shows a similar temporal profile of the ASE, with an input energy (4 mJ) well above the threshold, which leads to relaxation oscillations with spikes of 600 ps pulse-width and separation of 1 ns. Such spikes, but of microsecond duration, are quite common in solid-state lasers such as in Nd-YAG or ruby [13]. It is important to emphasize that such relaxation oscillations are unsteady, with significant variations of the pulse shape and the spike profiles as shown in Figure 7.

**Figure 8:**
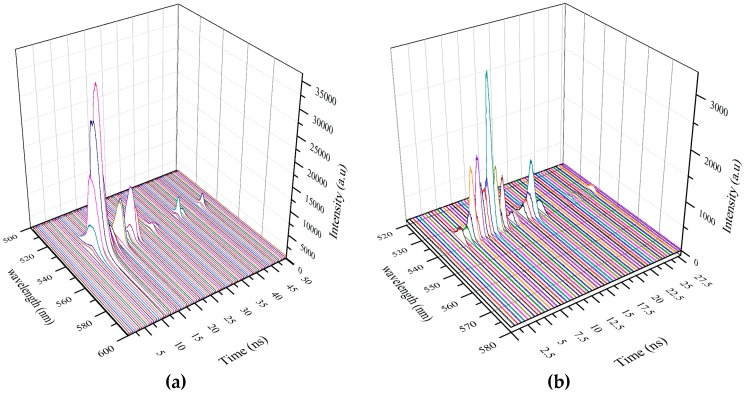
(**a**) Show the three dimensional (3D) ASE profile of GP under transverse excitation. It is displayed with wavelength (nm) in *x*-axis, Intensity (a.u) in *y*-axis and Time in *z*-axis, each fame represents a time duration of 0.5 ns. Note: ASE starts at *z* = 6 ns and reaches a maximum at 8 ns; but rapidly falls in next 500 ps. (**b**) Show the 3D representation of non-reproducible spikes of relaxation oscillation.

To get a better insight into the excited state dynamics of the conjugated polymer, the ASE pulses were captured in a Picomax spectrometer. This shows the intensity as a function of the wavelength (nm) with the time in ns in the *z*-axis. As shows in Figure 8a, the ASE starts at z = 6 ns, reaches a maximum at 8 ns, thereafter falling rapidly in the next 500 ps, and builds up for the next secondary pulse after 3 ns. Figure 8b is a representation of another relaxation pulse, indicative of the unsteady fluctuating conditions, common in relaxation oscillations.

**Figure 9:**
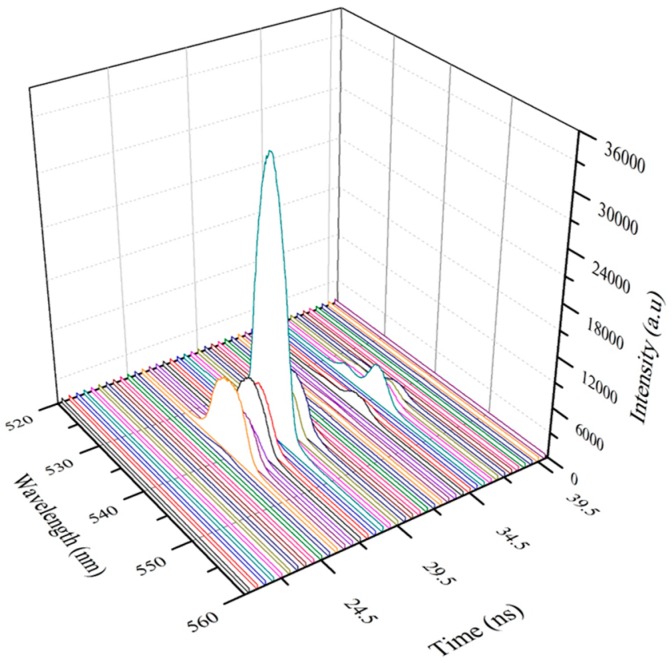
Show the 3D ASE profile of GP under longitudinal excitation. One could see that the spectral width is broader than transverse excitation, due to exponential attenuation of pump pulse, still the power is sufficient to produce relaxation oscillation (Note that different color lines are used to differentiate one time frame from the other).

Figure 9 is a representation of relaxation oscillations in the ASE of the longitudinal mode of excitation. In this case, both the spectral width and the intensities of the ASE pulses are weaker than those present in the transverse mode excitations. This is because of the strong exponential attenuation of the pump beam in the longitudinal mode.

As we have mentioned earlier, relaxation oscillation is the manifestation of the dynamic interaction between the excess population inversion and the abundant photon flux. With proper care of the pumping configuration and the operational parameters, it is possible to obtain single pulses of 500 ps or even lesser duration that could be extracted and subsequently amplified.

## 4. Conclusions

In this paper, we have reported the spectral properties of the green conjugated polymer [(9,9-dioctyl-2,7-divinylenefluorenylene)-*alt*-*co*-{2-methoxy-5-(2-ethylhexyloxy)-1,4-phenylene}] in a few Fbeam at 540 nm with moderate efficiency. More importantly, this paper presents a clear demonstration of a relaxation oscillation and a picosecond pulse generation from this new class of laser materials.

## Figures and Tables

**Table 1:** The Table shows the spectral features such as optical density, absorption peak, emission peak, stokes shift (cm^−1^) and quantum yield of GP in few solvents such as toluene and benzene (concentration = 500 nM).

Solvent	Optical Density (a.u)	Absorption Peak (nm)	Emission Peak (nm)	Stokes Shift (cm^−1^)	Quantum Yield
Benzene	1.07	481	505	988	0.75
Chloroform	1.57	480	507	1066	0.47
Toluene	1.48	481	505	988	0.49
THF	1.91	481	505	988	0.38
Dichlorobenzene	1.28	488	513	998	0.78
